# Interactome Profile of the Host Cellular Proteins and the Nonstructural Protein 2 of Porcine Reproductive and Respiratory Syndrome Virus

**DOI:** 10.1371/journal.pone.0099176

**Published:** 2014-06-05

**Authors:** Li Wang, Lei Zhou, Han Zhang, Yan Li, Xinna Ge, Xin Guo, Kangzhen Yu, Hanchun Yang

**Affiliations:** 1 Key Laboratory of Animal Epidemiology and Zoonosis of Ministry of Agriculture, College of Veterinary Medicine and State Key Laboratory of Agrobiotechnology, China Agricultural University, Beijing, People’s Republic of China; 2 The Ministry of Agriculture of the People’s Republic of China, Beijing, People’s Republic of China; The Ohio State University, United States of America

## Abstract

The nonstructural protein 2 (NSP2) is considered to be one of crucial viral proteins in the replication and pathogenesis of porcine reproductive and respiratory syndrome virus (PRRSV). In the present study, the host cellular proteins that interact with the NSP2 of PRRSV were immunoprecipitated with anti-Myc antibody from the MARC-145 cells infected by a recombinant PRRSV with 3xMyc tag insertion in its NSP2-coding region, and then 285 cellular proteins interacting with NSP2 were identified by LC-MS/MS. The Gene Ontology and enriched KEGG Pathway bioinformatics analyses indicated that the identified proteins could be assigned to different subcellular locations and functional classes. Functional analysis of the interactome profile highlighted cellular pathways associated with infectious disease, translation, immune system, nervous system and signal transduction. Two interested cellular proteins–BCL2-associated athanogene 6 (BAG6) and apoptosis-inducing factor 1 (AIF1) which may involve in transporting of NSP2 to Endoplasmic reticulum (ER) or PRRSV-driven apoptosis were validated by Western blot. The interactome data between PRRSV NSP2 and cellular proteins contribute to the understanding of the roles of NSP2 in the replication and pathogenesis of PRRSV, and also provide novel cellular target proteins for elucidating the associated molecular mechanisms of the interaction of host cellular proteins with viral proteins in regulating the viral replication.

## Introduction

Porcine reproductive and respiratory syndrome (PRRS) is an important swine disease, causing great economic losses to the swine industry worldwide [1], [2]. This disease was first described as “mystery swine disease” in the United states in 1987 [3], which is characterized by severe reproductive failure in sows and respiratory disorders in all age of pigs [3], [4]. In 2006, atypical PRRS outbreak caused a dramatic decline of pig breeding stock amount and huge economic losses to pig production in China [5]. Porcine reproductive and respiratory syndrome virus (PRRSV), the causative agent of this disease, is an enveloped, single-stranded positive sense RNA virus, which belongs to the order *Nidovirales*, family *Arteriviridae*, genus *Arterivirus* [6], [7]. The viral genome is approximately 15 kb in length and encodes at least ten open reading frames (ORFs) comprising of ORF1a, ORF1b, ORF2a, ORF2b, ORFs3-7 and the recently discovered ORF5a [8], [9]. ORF1a and ORF1b comprised approximately two-third of the genome encode the replicase polyproteins which are considered to be autoproteolytically cleaved into at least 14 non-structural proteins (NSPs) including the newly discovered transframe fusion (TF) in NSP2-coding region [10], [11].

Among the 14 NSPs of PRRSV, NSP2 is the largest replication protein with three major domains–an N-terminal papain-like cysteine protease domain (PLP2), a functionally unspecified middle hypervariable region and a C-terminal transmembrane (TM) domain [12], [13]. A number of studies have indicated that the middle hypervariable region of the NSP2 is able to tolerate foreign gene insertion and deletion [12], [14], [15]. As well, it has been verified that the atypical PRRS outbreak in China was caused by a highly pathogenic PRRSV (HP-PRRSV) strain with 30-amino-acid deletion in this coding region [15], [16]. NSP2 was also recognized to play an important role in the PRRSV replication, in which the PLP2 possesses both trans- and cis-cleavage activities involving in viral replicase polyprotein processing that is cleaved at the NSP2–NSP3 (NSP2/3) junction [17]–[19]. In addition, the NSP2 of arterivirus can collaborate with NSP3 to form the double-membrane vesicles (DMVs), which are derived from paired endoplasmic reticulum (ER) membranes, and these DMVs provide the sites for viral RNA synthesis [20]–[22]. The PLP2 of NSP2 was also considered to belong to the ovarian tumor domain (OTU)-containing superfamily and possess the deubiquitinating (DUB) and deISGylating activities to inhibit Ub- and ISG15-dependent antiviral pathways [23]–[26]. During the past years, a number of studies have provided evidence that protein ubiquitylation plays an important role in the regulation of both innate and adaptive immune system, and meanwhile, some viruses take advantage of the OTU domain-containing proteases to regulate Ub-dependent innate immunity via its DUB protease activity [27], [28]. Previous studies showed that the NSP2 of PRRSV can exploit its DUB potential to inhibit NF-κB and RIG-mediated innate immune signaling [8], [25], [29], and the loss of DUB activity may strikingly enhance innate immune signaling [30]. Thus, these findings reveal that NSP2 not only is a critical factor in the virus replication, but also can involve in the virus evasion from innate immune. However, the molecular mechanisms of its involvement in these functions have not been elucidated clearly.

Given the important role of the NSP2 in arterivirus replication and viral innate immune evasion,we employed the immunoprecipitation technique coupled with the LC-MS/MS and bioinformatics analysis to explore and characterize the host cellular proteins interacting with PRRSV NSP2 and regulating its function. Furthermore, we generalized the characteristics of the cellular proteins interacting with NSP2 to generate an interactome profile of NSP2, in order to understand the mechanism of arterivirus replication and its immune evasion from host antiviral defense.

## Materials and Methods

### Ethics Statement

The animal use in this study was approved by The Laboratory Animal Ethical Committee of China Agricultural University. The animal treatment was performed according to the Chinese Regulations of Laboratory Animals and the Guidelines for the Care of Laboratory Animals (Ministry of Science and Technology of People’s Republic of China), and GB 14925-2010 Laboratory Animal-Requirements of Environment and Housing Facilities (National Laboratory Animal Standardization Technical Committee).

### Cells, Infectious cDNA Clone Plasmid and Virus

The African green monkey kidney epithelial cell line MARC-145 cells, human embryonic kidney 293FT cells and baby hamster kidney cell line BHK-21 cells were cultured in Dulbecco’s modified eagle medium (DMEM) (Invitrogen Corporation, Carlsbad, CA) containing 10% fetal bovine serum (FBS, Hyclone Laboratories, Inc., South Logan, UT) at 37°C, with 5% CO_2_. A highly pathogenic PRRSV JXwn06 and its full-length infectious cDNA clone plasmid (pWSK-JXwn) were used in this study [15]. The plasmid was used as the backbone for the insertion of Myc tag.

### Plasmid Construction

A six-week-old SPF landrace piglet was obtained from Beijing Center for SPF Swine Breeding & Management. The piglet was euthanized and necropsied, and its thymus and liver tissues were collected. Coding region fragments of BAG6 and AIF1 were amplified from the total RNA of pig thymus and liver tissues by RT-PCR using the designed primers based on the sequences available in GenBank (NM_001145382.1, BAG6; XM_003135370.2, AIF1), respectively, and the amplified products were extracted using a RNAprep Pure Tissue Kit (TIANGEN, Beijing), according to the manufacturer’s protocols. The reverse transcriptions were performed by using M-MLV reverse transcriptase (Promega, Madison, WI) in a reaction system with total volume of 20 µl. The NSP2 gene of PRRSV was amplified by PCR using pWSK-JXwn as templates. CMV-FLAG-BAG6, CMV-FLAG-AIF1 and pCMV-HA-NSP2 were constructed by conventional techniques. All the primers used in this study are listed in Table 1.

**Table 1:** Primers used in this study.

Primer^a^	Sequence (5′-3′)^b^	Use
Myc-**F**	GAGCAGAAACTCATCTCTGAAGAAGATCTGGAACAAAAGTTGATTTCAGAAGAAGATCTG	Amplification of 3xMyc tag gene
Myc-**R**	CAGATCTTCCTCAGAGATGAGCTTCTGTTCCAGATCTTCTTCTGAAATCAACTTTTGTTCC	Amplification of 3xMyc tag gene
NSP2-Myc-L-**F**	**CAATTG** CGAGCAGAAACTC	Amplification of Myc-fragment A
NSP2-Myc-L-**R**	GGACATGAGCCCCAGATCTTCCTCAGAGATG	Amplification of Myc-fragment A
NSP2-Myc-R-**F**	GAGGAAGATCTGGGGCTCATGTCCACTGGAC	Amplification of Myc-fragment A
NSP2-Myc-R-**R**	AGCCTCACGCATGAT**GCTGAGG**CATGC	Amplification of Myc-fragment A
BAG6-**F**	CTGATAACTTAATCTTCGTGC	Amplification of BAG6 gene
BAG6-**R**	AACAACTAGGGATCTTCAGC	Amplification of BAG6 gene
CMV-BAG6-**F**	CCC**AAGCTT**ATGGAGCCCAATGATAGTACC	Construction of CMV-FLAG-BAG6
CMV-BAG6-**R**	GGA**AGATCT**CTAGGGATCTTCAGCAAAGGC	Construction of CMV-FLAG-BAG6
AIF1-**F**	AAATGTTCCGGTGTGGAGG	Amplification of AIF1 gene
AIF1-**R**	TTCAGTCCTCATGAATGTTGAAG	Amplification of AIF1 gene
CMV-AIF1-**F**	CCC**AAGCTT**ATGTTCCGGTGTGGAGGCTTGG	Construction of CMV-FLAG-AIF1
CMV-AIF1-**R**	CGG**GAATTC**TCAGTCCTCATGAATGTTGAAGAGC	Construction of CMV-FLAG-AIF1
W4**F**	TGAGCCTCTGGATTTGTCTGC	Detection of pWSK-Myc-JXwn
W4**R**	GGCGATCTATTAGGAGCAGTT	Detection of pWSK-Myc-JXwn
NSP2-**F**	CGC**GTCGAC**GGCCGGAAAGAGAGCAAGGA	Construction of pCMV-HA-NSP2
NSP2-**R**	GA**AGATCT**TCATCCCCCTGAAGGCTTCGAA	Construction of pCMV-HA-NSP2

a Boldface F denotes a forward PCR primer; Boldface R denotes a reverse PCR primer.

b Restriction sites introduced by PCR are shown in boldface.

### Construction of a Chimeric Infectious cDNA Clone with a 3xMyc Tag Inserted into the NSP2 Hypervariable Region

To label the NSP2 with a 3xMyc tag, the region from NSP2 aa 338 to aa 367 was replaced by the 3xMyc tag fragment (30 aa). The 3xMyc tag fragment was first obtained by the overlapping extension PCR with the primer Myc-F and Myc-R (Table 1). Then the NSP2 overlapping region was added to the 3xMyc fragment by PCR with the primers NSP2-Myc-L-F and NSP2-Myc-L-R, meanwhile, partial NSP2 fragment with an overlapping region with 3xMyc fragment was amplified by using the pWSK-JXwn as templates with the primers NSP2-Myc-R-F and NSP2-Myc-R-R (Table 1). Finally the fusion PCR was performed and the obtained fusion fragment was excised with *Mfe* I and *BbvC* I, in order to clone back to the fragment A of pWSK-JXwn to generate the plasmid Myc-fragment A. The Myc-fragment A was then inserted into the pWSK-JXwn backbone as described previously [15] to generate a recombinant clone plasmid pWSK-Myc-JXwn.

### Recovery and Identification of the Chimeric Virus

In vitro transcription and transfection were performed as described previously [15]. The chimeric full-length cDNA clone pWSK-Myc-JXwn was linearized by cleavage with restriction enzyme *Pac* I, followed by *in vitro* transcription with mMessage high-yield capped RNA transcription kit (Ambion, Austin, TX) and then the purified RNA was transfected into BHK-21 cells by using DMRIE-C reagent (Invitrogen Corporation, Carlsbad, CA). The transfected cells were incubated for 24 h, and then the cell culture supernatants were harvested and passaged in MARC-145 cells serially.

The rescued viruses and the stability of 3xMyc tag in the NSP2-coding region were identified by confocal microscopy analysis with an anti-PRRSV N monoclonal antibody SDOW17 (Rural Technologies, Inc., Brookings, SD) and an anti-Myc polyclonal antibody (Sigma, St. Louis, MO). To further detect PRRSV, the RNAs of the fifth and tenth passage of the chimeric viruses were extracted from cell culture supernatants by using a QIAamp viral RNA kit (Qiagen, Chatsworth, CA) and RT-PCR was then performed with the primer pairs W4F/R (Table 1) [16]. The PCR products were sequenced to check the existence of 3xMyc tag in the NSP2-coding region.

To compare the growth ability of chimeric virus with its backbone parental virus, the MARC-145 cells monolayer in T-25 flasks were infected with the fifth passage of the chimeric virus and the parental virus at a multiplicity of infection (MOI) of 0.01, respectively. The supernatants were collected at different time points post-inoculation (pi) and the virus titers were determined by the microtitration infectivity assay and recorded as TCID_50_ per milliliter by using the Reed-Muench method.

### Confocal Microscopy Analysis

MARC-145 cells were seeded on coverslips in 24-well plates and cultured, then infected with RvMyc-JXwn and RvJXwn at a MOI of 0.01 respectively. At 48 h post-infection, the cells were fixed with 4% paraformaldehyde for 30 min, followed with being permeabilized with 0.1% Triton X-100 for 15 min and blocked with 5% bovine serum albumin (BSA) for 30 min at room temperature. The cells were then incubated with both anti-Myc polyclonal antibody and anti-PRRSV N monoclonal protein antibody SDOW17 (1∶200) for 2 h at 37°C. After being washed with phosphate-buffered saline (PBS) for three times, the cells were stained with TRITC-conjugated goat anti-rabbit and FITC-conjugated goat anti-mouse secondary antibodies (Beyotime, Nanjing, Jiangsu) for 1 hour at 37°C. Followed by washing three times with PBS, the coverlips were mounted and observed under the Olympus BX61 confocal microscope.

### Detecting the Expression of NSP2

MARC-145 cells in 6-well plates were infected with the chimeric virus and the parental virus at a MOI of 0.01, respectively. Then the cells were collected at different time points (12 h to 60 h post-infection). The samples were then subjected to Western blot with anti-Myc antibody.

### Immunoprecipitation and Co-immunoprecipitation

For immunoprecipitation (IP), RvMyc-JXwn- and RvJXwn-infected MARC-145 cells were lysed in IP buffer (Beyotime, Nanjing, Jiangsu) and incubated at 4°C on a shaker for 30 min, followed by centrifugation at 12,000 g for 20 min. A total of 600 µl of the supernatants at a final concentration of 3 µg/µl were precipitated with anti-Myc monoclonal antibody (Sigma, St. Louis, MO) in conjunction with Protein G Sepharose 4 Fast Flow (GE Healthcare Bio-Science, Piscataway, NJ) and were incubated with gentle rocking overnight at 4°C. The beads were washed five times with cold IP buffer and boiled with 5xSDS loading buffer for 5 min, followed by SDS-PAGE and silver staining or Western blot. For co-immunoprecipitation (Co-IP), the 293FT cells were co-transfected with BAG6- or AIF1-expressing plasmids CMV-FLAG-BAG6/CMV-FLAG-AIF1 and NSP2- expressing plasmid pCMV-HA-NSP2, and meanwhile, the cells co-transfected with empty vector pCMV-HA or CMV-FLAG were served as controls. Plasmid-transfected cells were lysed in IP buffer at 24 to 36 h post-transfection. The cell lysates were precipitated with appropriate antibodies in conjunction with beads as described above. The immunoprecipitated proteins were detected by Western blot.

### Silver Staining and Mass Spectrometric Identification of Proteins

The immunoprecipitated proteins were fractionated by electrophoresis on 8% and 15% SDS-PAGE gels and the gels were stained using a Silver Stain kit for Mass Spectrometry (Thermo, Rockford, IL) according to the manufacturer’s protocols. Prestained Protein Ladder (Thermo) was used for estimating the approximate sizes of separated proteins. All distinct bands in the lane of RvMyc-JXwn-infected group and the gel at parallel area in the lane of RvJXwn-infected group were excised and subjected to LC-MS/MS as described previously [31]. Briefly, gel pieces were distained with 50% acetonitrile/50 mM NH_4_HCO_3_ and then washed with water. After being shrunk with acetonitrile, the gel pieces were reduced with 10 mM of DTT (60°C, 30 min), followed by alkylation with 55 mM Iodoacetamide (in dark, 25°C, 30 min). After being washed with water and shrunk with acetonitrile again, the gels were incubated with trypsin (10 ng/µl) overnight at 37°C. Peptides were extracted with 50% acetonitrile/5% formic acid at 37°C for 2 h. Peptides were separated using a nano-flow HPLC (Easy nanoLC, Thermo Fisher Scientific, Bremen, Germany). The 15-cm reverse-phase column (inner diameter 75 µm, 3 µm, C18) was in house made. The HPLC was coupled to an LTQ-Orbitrap mass spectrometer (Thermo Fisher Scientific, Bremen, Germany). Mass spectra were acquired in a data-dependent manner, with an automatic switch between MS and MS/MS scans. MS spectra were acquired in the Orbitrap analyzer, with a mass range of 300–2000 and a target value of 10^6^. Up to the 8 most intense ions in each full MS scan were fragmented with the CID (collision energy 35%) method, and MS/MS spectra were acquired in the LTQ analyzer and a target value of 30 000, with singly charged ions excluded.

### Bioinformatics Analysis

The functional annotation and classification of all the proteins were determined by using BlAST2GO program [32] against the non-redundant protein database (nr) at NCBI and KEGG pathway database [33]. The Protein-Protein interact network was performed by using the Cytoscape software [34].

### Western Blot Analysis

Protein samples were separated by electrophoresis on 8% (w/v) SDS-PAGE and transferred to PVDF membranes (Millipore, Bedford, MA). After blotting, the membranes were probed with appropriate antibodies. Subsequently, the membranes were washed ten times with 0.05% PBST and incubated with horseradish peroxidase (HRP) conjugated goat-anti-mouse or goat-anti-rabbit IgG (Beyotime, Nanjing, Jiangsu). The enhanced chemiluminescence (ECL) system (Vigorous, Beijing) was utilized to detect the blotted proteins.

## Results

### Recovery of 3xMyc-taged PRRSV

**Figure 1:**
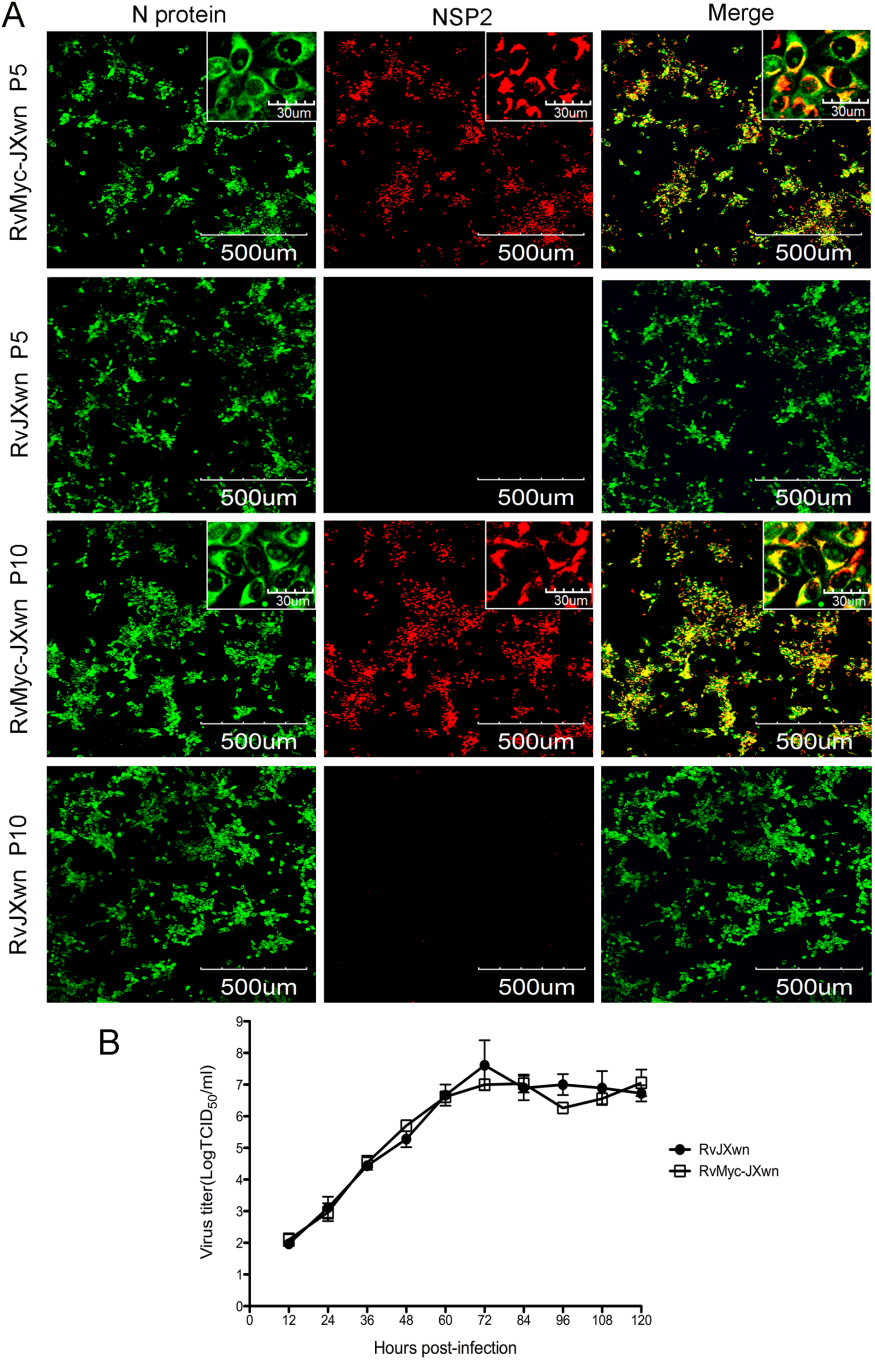
Identification of the rescued recombinant PRRSV with the 3xMyc-tag. (A) The confocal microscopy analysis of fifth passage of the recombinant virus RvMyc-JXwn and its parental virus RvJXwn of the passage 5 and 10 with anti-PRRSV N protein monoclonal antibodies (SDOW17) and anti-Myc polyclonal antibody. (B) *In vitro* growth kinetics of the fifth passage of the recombinant virus RvMyc-JXwn and its parental virus RvJXwn were drawn by assaying the viral titers of the supernatants harvested from 12 h to 120 h post-infection using microtitration infectivity assays. Data are means±standard deviations (error bars) from three independent trials. No significant difference between the recombinant virus and the parental virus (*p*>0.05).

The region–aa 324 to aa 434 of PRRSV NSP2 has been shown to be dispensable for viral replication [12]. In order to label the NSP2 and facilitate to identify the host cellular proteins interacted with NSP2, the region–aa 338 to aa 367 of PRRSV JXwn06 NSP2 was accordingly replaced with 3xMyc tag in the infectious clone plasmid pWSK-JXwn. Together with parental backbone plasmid pWSK-JXwn, the new generated Myc-labeled full-length plasmid pWSK-Myc-JXwn was linearized and transcribed *in vitro*. Then the transcribed capped RNAs were transfected into BHK-21 cells, and the supernatant obtained from BHK-21 cells at 24 h post-transfection were serially passaged in MARC-145 cells. CPE typical of PRRSV in MARC-145 was observed at the first passage of both cDNA clones. To confirm the successful recovery of virus and examine the stability of the foreign tag in the virus, the chimeric virus designated as RvMyc-JXwn and its parental virus RvJXwn were serially passaged in MARC-145 cells, and then the confocal microscopy analysis (Figure 1A) with Myc polyclonal antibody and PRRSV N protein monoclonal antibody and the sequence analysis of the NSP2-coding region including Myc tag gene were performed. The confocal microscopy analysis demonstrated that Myc and N protein were present in the cells, and the Myc revealed a typical perinuclear localization pattern like NSP2 possessing as described previously [12], [35], [36]. Both the confocal microscopy analysis (Figure 1A) and the sequence analysis (data not shown) showed that, the chimeric virus was rescued successfully and the foreign 3xMyc tag could be stably maintained in the virus for at least 10 passages *in vitro*.

To further analyze whether the insertion of 3xMyc tag influences the replication of chimeric virus, the growth kinetics of the fifth passage of RvMyc-JXwn was compared with its parental backbone virus RvJXwn in MARC-145 cells. As shown in Figure 1B, the chimeric virus RvMyc-JXwn displayed similar growth kinetics to the parental virus RvJXwn, suggesting that the replacement of 30-aa region of NSP2 with an equal-length foreign 3xMyc tag has no effect on PRRSV replication. Thus, we performed subsequent experiments by using the fifth passage of chimeric virus RvMyc-JXwn and its parental strain RvJXwn.

### The Expression of NSP2 upon PRRSV Infection

**Figure 2:**
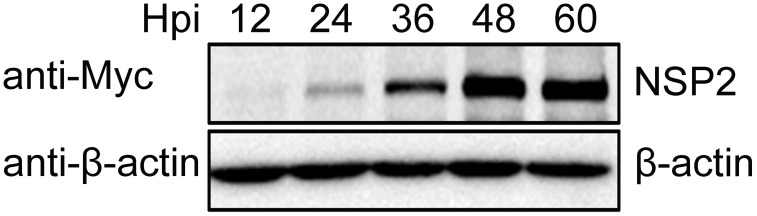
The expression of PRRSV NSP2. Cell lysates from RvMyc-JXwn-infected MARC-145 cells at different time points were subjected to Western blot with anti-Myc antibody and anti-β-actin antibody.

To detect the expression of NSP2, the MARC-145 cells infected with the chimeric virus RvMyc-JXwn and the parental virus RvJXwn were collected at 12 h to 60 h post-infection, then the samples were subjected to Western blot with anti-Myc antibody, and the expression of β-actin was served as a reference. The results showed that the expression level of NSP2 increased during PRRSV infection, and reached a peak at 48 h post-infection (Figure 2). Thus, we collected the samples at 48 h post-infection for subsequent interactome analyses.

### Identification of Host Cellular Proteins that Interact with the NSP2 of PRRSV

**Figure 3:**
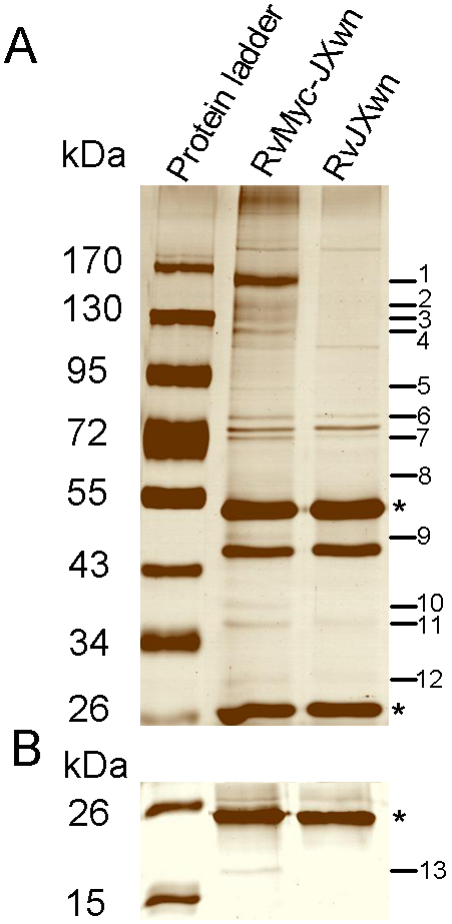
Identification of the cellular proteins that interact with PRRSV NSP2 by immunoprecipitation (IP). Cell lysates from RvMyc-JXwn- or RvJXwn-infected MARC-145 cells were immunopreciptiated with anti-Myc antibody, and subsequently the immunoprecipitated proteins were separated both by 8% (A) and 15% (B) SDS-PAGE and visualized by sliver staining. Asterisks indicate the protein bands of IgG heavy chain with 55 KDa or IgG light chain with 26 KDa. The numbers on the right lane show the differential protein bands between RvMyc-JXwn- and RvJXwn-infected MARC-145 cells.

To efficiently precipitate NSP2 from PRRSV-infected cells and subsequently identify the host proteins that interact with NSP2, MARC-145 cells were infected with the rescued virus RvMyc-JXwn or parental viruses RvJXwn at 0.01 MOI, respectively. Infected cells harvested at 48 h post-infection were immunopricicipitated with monoclonal antibody against Myc. The immunoprecipitated proteins were resolved in 8% and 15% SDS-PAGE respectively and visualized by sliver staining. Compared with RvJXwn, at least 13 bands of proteins specifically precipitated in Myc-tagged RvMyc-JXwn were detected (Figure 3). 617 cellular proteins were identified by further LC-MS/MS analysis on these protein bands apart from the background, which were identified from the 13 gel area in the lane of RvJXwn infection group. Among them, 285 proteins with high Confidence Icons (*p*<0.01) were used for bioinformatics analysis. A summary of these proteins interacting with NSP2 at 48 h following PRRSV infection are given in (Table S1), with their protein scores and sequence coverage.

### Functional Analyses of Identified Proteins

**Figure 4:**
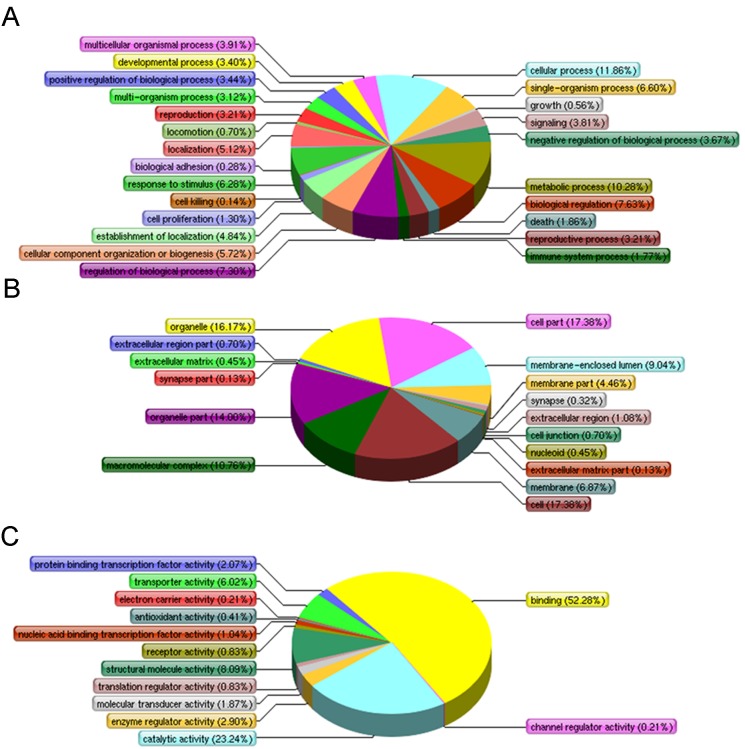
The annotation of proteins interacting with PRRSV NSP2 using Gene Ontology. (A) Biological process. (B) Cellular components. (C) Molecular function.

To reveal functional insights into the interactome of NSP2, 285 identified proteins were assigned for bioinformatics analyses. The results indicated three main types of annotations including biological process, cellular components and molecular functions were obtained from the gene ontology consortium website (Figure 4). Subclasses associated with cellular process (11.86%), metabolic process (10.28%), biological regulation (7.63%), biological process regulation (7.30%), single-organism process (6.60%) and response to stimulus (6.28%) were enriched in biological process category (Figure 4A). The most enriched subclasses in cellular component included cell (17.38%) and cell part (17.38%), organelle (16.17%) and organelle part (14.00%), macromolecular complex (10.76%), membrane-enclosed lumen (9.04%) (Figure 4B). The enrichment based on molecular function showed binding (52.28%), catalytic activity (23.24%), structural molecule activity (8.09%) and transporter activity (6.02%) (Figure 4C). A more detailed summary containing the GO annotation for individual protein is provided in (Table S2).

**Figure 5:**
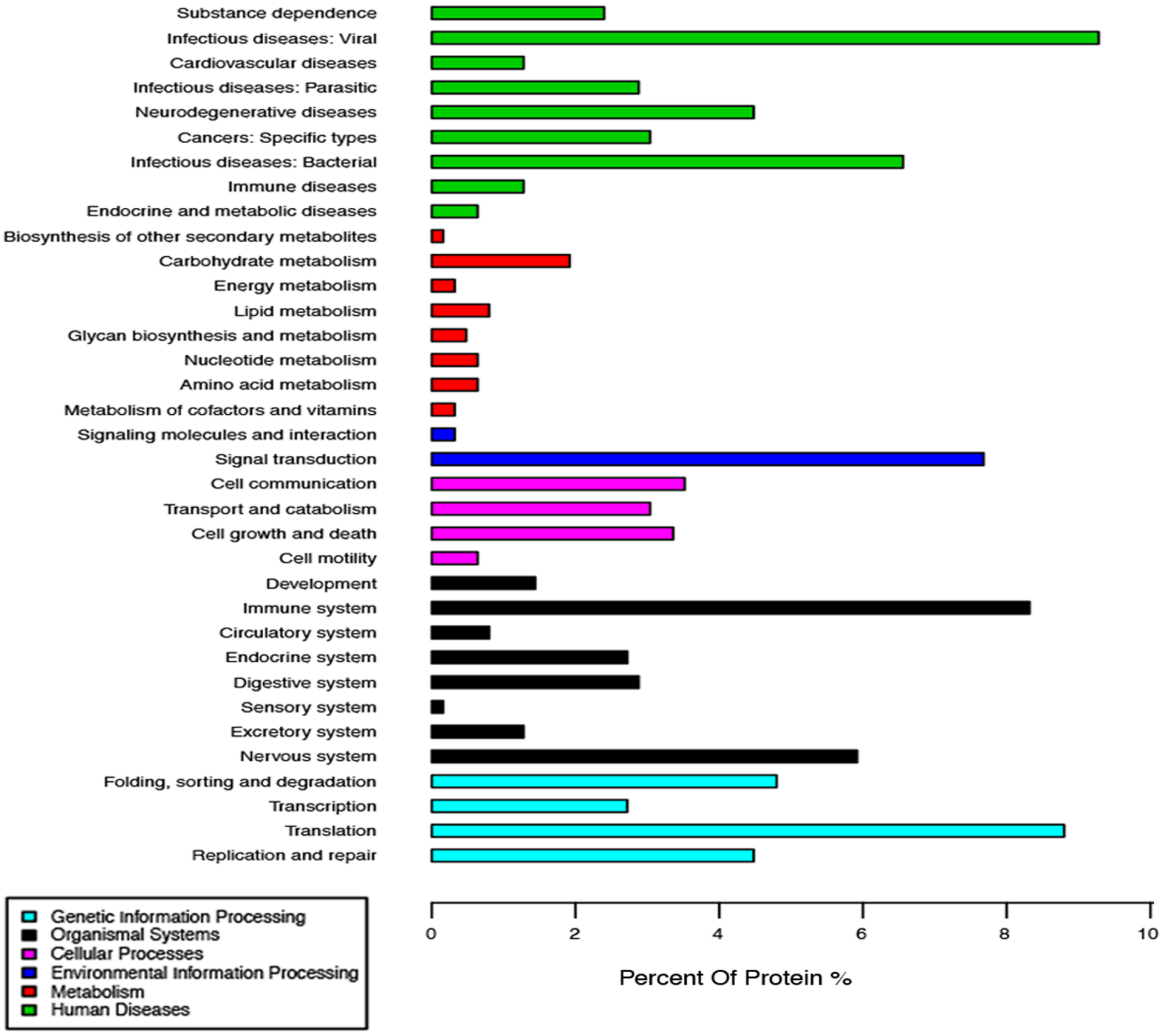
Classification of the enriched KEGG Pathways of the cellular proteins interacting with PRRSV NSP2.

In addition, the pathway analysis of infection network based on KEGG revealed an enrichment of 167 pathways (Table S3). The more prominent pathways involved in infectious disease (117 proteins), translation (55 proteins), immune system (52 proteins), signal transduction (48 proteins), nervous system (37 proteins), replication and repair (28 proteins), cell communication (22 proteins), cell growth and death (21 proteins) (Figure 5 and Table S3).

**Figure 6:**
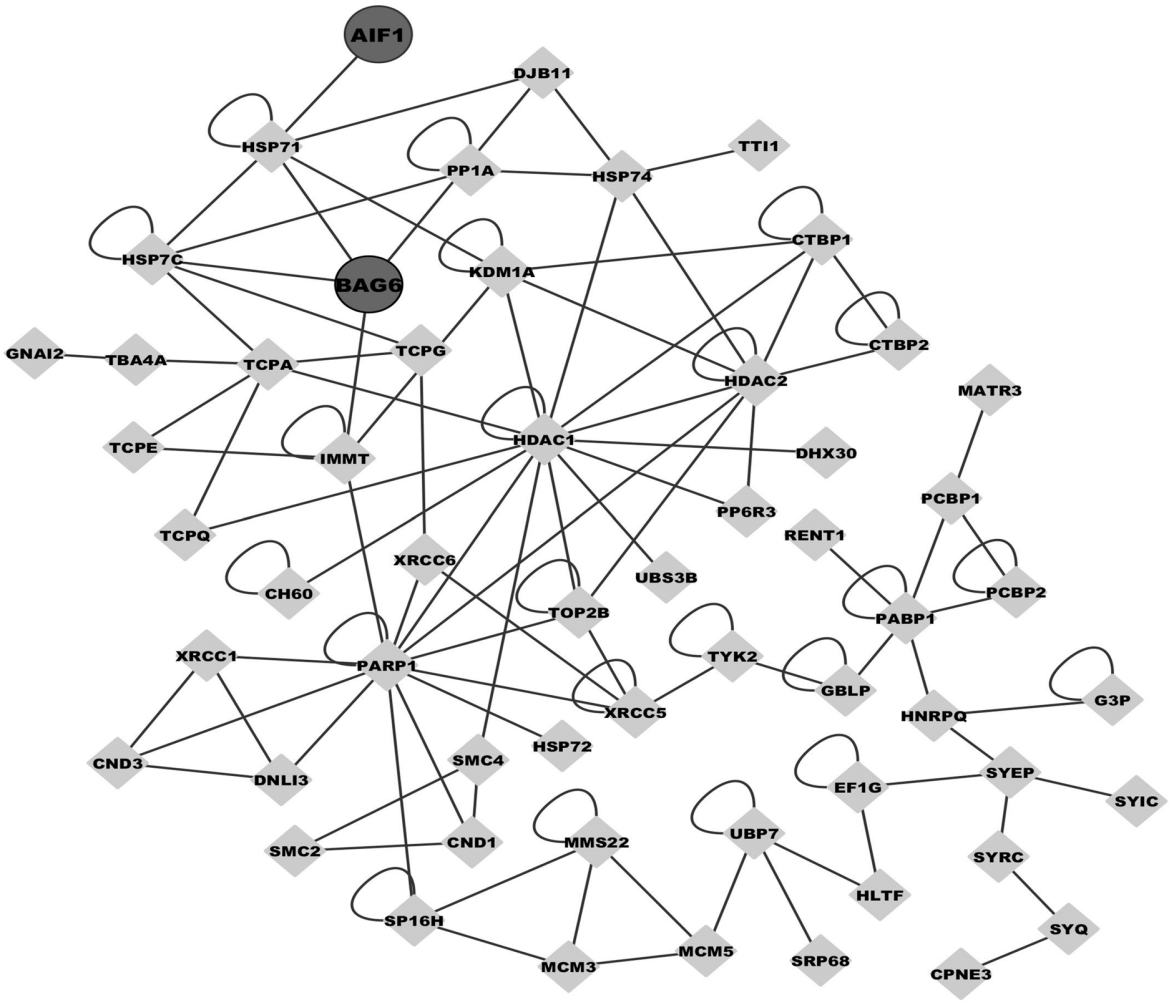
The interaction network of the identified proteins with BAG6 and AIF1.

Considering the ER-distribution and recognized functions of NSP2 during PRRSV infection in host cell, we further focused on the BAG6 and AIF1, the two novel host cellular proteins that interact with NSP2 identified in this study. The interactome profile of the identified proteins associated with BAG6 and AIF1 are shown in Figure 6.

### Validation of the Proteins that Interact with the NSP2 of PRRSV by Western Blot Analysis

**Figure 7:**
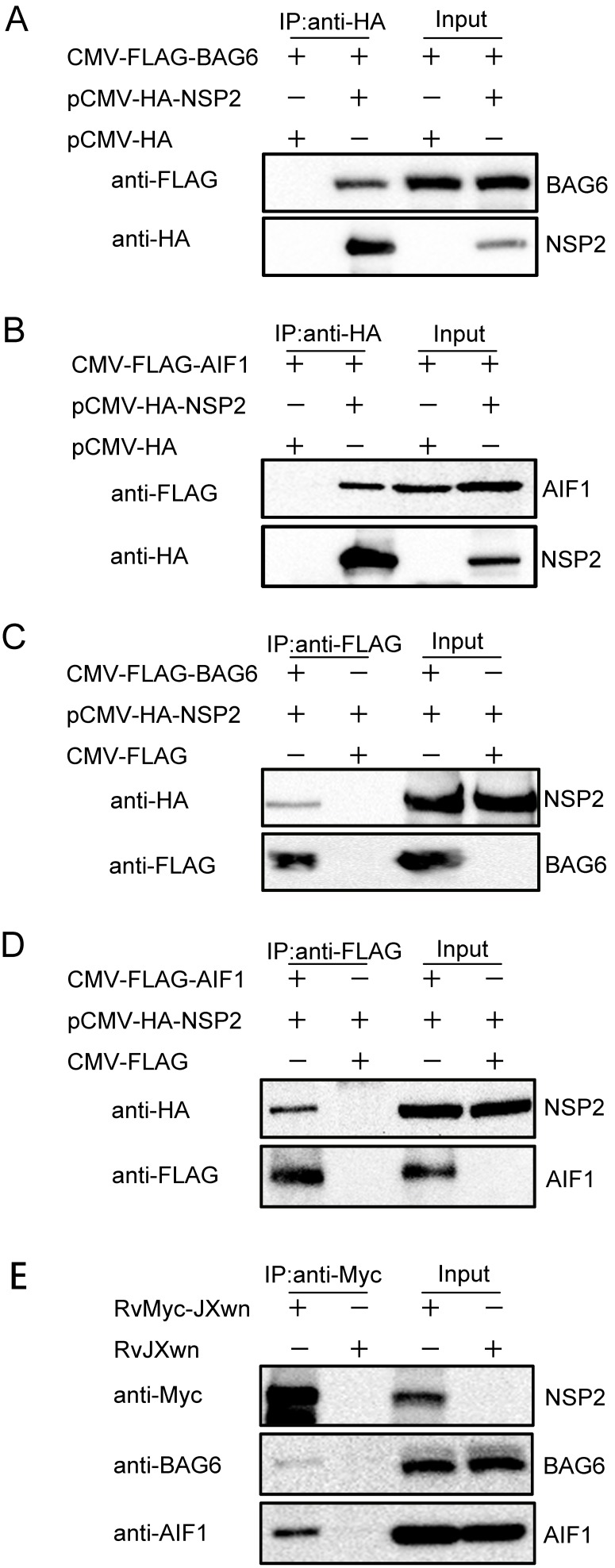
Confirmation of the interaction of PRRSV NSP2 with BAG6 and AIF1. (A to D) The interaction of NSP2 and exogenous BAG6 (A, C) and AIF1 (B, D). 293FT cells were co-transfected with 6 µg of the indicated plasmids. Cell lysates were prepared at 24 h after transfection and the proteins were immunoprecipitated with anti-HA or anti-FLAG antibodies. Proteins in cell lysates (input) and immunoprecipitated samples were detected with the antibodies against FLAG and HA by Western blot. (E) The interaction of NSP2 with endogenous BAG6 and AIF1. MARC-145 cells were infected with the recombinant virus RvMyc-JXwn and its parental virus RvJXwn. Cell lysates were prepared at 48 h post-infected and subjected to IP with anti-Myc antibody, the immunoprecipitated samples were detected with the antibodies against BAG6 and AIF1 by Western blot.

The cellular proteins BAG6 and AIF1 were detected in the L1 and L7 (Figure 3A). The amplified cDNAs of porcine BAG6 and AIF1 genes were sequenced, indicating a new transcript splice variant with a 108-nt insertion in porcine BAG6. The sequences of porcine BAG6 and AIF1 genes have been submitted to GenBank (accession nos: KF941298, KF941299). Three plasmids–CMV-FLAG-BAG6, CMV-FLAG-AIF1 and pCMV-HA-NSP2 were constructed. After 293FT cells were transfected with pCMV-HA-NSP2 or empty vector (pCMV-HA) along with the FLAG-tagged BAG6-expressing plasmid (CMV-FLAG-BAG6) or FLAG-tagged AIF1-expressing plasmid (CMV-FLAG-AIF1), Co-IP was performed with anti-HA antibody. And then the immune-complexes were resolved in 8% SDS-PAGE and probed for the presence of BAG6 or AIF1 by using anti-FLAG antibody. The results showed that Both BAG6 and AIF1 could be readily detected only in the presence of NSP2, but not in the presence of empty vector (Figure 7A and B). The similar results were obtained in another Co-IP experiment by using anti-FLAG antibody (Figure 7C and D). In order to further confirm the interaction between these two host proteins and NSP2, the MARC-145 cells were infected with RvMyc-JXwn and RvJXwn respectively, and then the endogenous BAG6 and AIF1 that interact with NSP2 were examined by using IP with anti-Myc antibody. The endogenous BAG6 and AIF1 could be detected in RvMyc-JXwn-infected cells, but not in RvJXwn-infected cells (Figure 7E). These results confirmed that PRRSV NSP2 were able to interact with the cellular proteins BAG6 and AIF1.

## Discussion

PRRSV has been recognized to be able to evade the host immune response and cause persistent infection [37], however the corresponding mechanisms are not fully elucidated. It is well known that virus can infect hosts and survive in host cells through interacting with the factors of the host cells, exploiting the cellular pathways and subverting defense systems established by host cells to inhibit viral propagation [38]–[41]. Therefore, elucidating the virus-host interactions is a considerable way for exploring the mechanisms associated with viral pathogenesis and host anti-virus response.

### Why did we Construct a Chimeric Virus to Identify the Host Proteins that Interact with the NSP2 of PRRSV?

The NSP2 of PRRSV is the largest nonstructural protein, as well as recognized as the most variable region in the genome of PRRSV, with a number of substitutions, insertions and deletions. NSP2 does not only perform the critical cleavage of NSP2/NSP3, but it is also a cofactor of NSP4-associated cleavage [42]. Moreover, the functions of NSP2 OUT domain on deconjugating ISG15 and an ubiquitin-like molecule in IFN pathway have been documented [24]. Importantly, with collaborated with NSP3, the NSP2 can form the double-membrane vesicles (DMVs) that provide the sites for viral RNA synthesis [43]. Considering PRRSV NSP2 shares the important functions associated with viral replication and pathogenicity, it is very valuable to investigate the interactome profile of NSP2 with the host cellular proteins.

In the present study, according to the NSP2’s genetically flexible properties [12], [14], [15], [18], the NSP2 was labeled with 3xMyc tag in the live virus by constructing a recombinant PRRSV–RvMyc-JXwn. Then the constructed chimeric virus was utilized to further investigate the direct and indirect interactive cellular proteins with NSP2 in PRRSV-infected MARC-145 cells. Compared with the classical immunoprecipitation by transfecting one single protein into the cells [44], [45], this method can present the native protein conformation during virus replication, as well as it can explore the cellular proteins that interact directly or indirectly under the presence of other viral proteins with NSP2, which are easily missed in the classical method. Otherwise, this method also can take the advantage of commercial Myc-antibody with higher affinity and specificity to improve the efficiency of immunoprecipitation.

### Functional Analysis of NSP2 Interaction Network

In this study, 285 host cellular proteins that interact with NSP2 were identified in RvMyc-JXwn-infected cells by the combined IP and LC-MS/MS. To further explore the biological significance of the interaction between NSP2 and host cellular proteins, we utilized bioinformatics analysis to comprehensively evaluate and characterize the identified proteins. All the 285 proteins could be assigned to different functional annotations and classifications. Notably, a significant proportion of the enriched KEGG pathways, including 20 enriched KEGG pathways and 117 proteins, were shown to be associated with the infectious disease (Figure 5 and Table S3). These results implicate that like others pathogens, PRRSV may exploit similar host cellular components and share a common or similar pathogenesis. Thus the researches about other pathogens can offer a way to study the pathogenesis of PRRSV.

The second highest proportion of enriched KEGG pathways was the pathways of translation, including 5 enriched KEGG pathways, 55 proteins. It is reasonable to find the translation pathways that were enriched during the PRRSV infection. After virus entry and release genome into the host cell’s cytoplasm, the translation process is initiated. Firstly, the PRRSV translate its two replicase proteins coded by ORF1a and ORF1b, by employing the host translation system, to yield the polyprotein precursors pp1a and pp1ab, resulting in the generation the replication/transcription complexes (RTCs) through autoproteolysis [11], [46], [47]. Following the synthesis of subgenomic RNA mediated by the RTCs, the viral structural proteins are translated from the subgenomic mRNA [11], [37], [46], [47]. Our data further suggest that NSP2 play an essential role in the replication of PRRSV.

Previous studies have demonstrated that PRRSV has a predilection for infecting immune cells and driving immunosuppression and the persistent infection in pigs [48]. However the mechanisms associated with the PRRSV-induced immunosuppression are still poorly understood. Our results revealed that immune system, including 14 enriched KEGG pathways and 52 proteins, and signal transduction pathways including 10 enriched KEGG pathways and 48 proteins, were enriched in the NSP2 interaction network, including antigen processing and presentation, B cell receptor and T cell receptor signaling pathway, chemokine signaling pathway, NOD-like receptor signaling pathway, RIG-I-like receptor signaling pathway, Toll-like receptor signaling pathway and NF-κB signaling pathway and so on (Table S3). PRRSV has been reported to infect porcine monocyte-derived DC (Mo-DC), and induce cell death and down-regulate the expression of MHC class I, MHC class II, CD11 b/c and CD14, resulting in the impairment of the antigen presentation ability of Mo-DC [49]. In addition, PRRSV infection can mediate apoptosis in B-cell and T-cell in lymphoid organs [50], but which proteins of PRRSV are involved in these phenomenon and its underlying mechanisms are still unknown. Our data hint that NSP2 may involve in these progress. A number of evidences have indicated that NSP2 is able to regulate NF-κB and RIG-mediated innate immune signaling pathway to suppress the host immune responses [24], [25], [29], [30]. Together with the data reported previously, our results provide an important clue for studying the NSP2 functions on the induction of immunosuppression and persistent infection induced by PRRSV.

Enriched Nervous system KEGG pathways including 10 enriched KEGG pathways and 37 proteins, and Neurodegenerative diseases KEGG pathways including 5 enriched KEGG pathways and 28 proteins were also found in the NSP2 interaction network. The nervous symptom can be observed in PRRSV-infected pigs, which was supported by some evidence in previous pathogenicity analysis showing that PRRSV infection could induce the central nervous system (CNS) lesions including lymphohistiocytic perivascular cuffing, gliosis and mild vasculitis [51], [52]. In addition, the PRRSV antigen can be detected in the cerebellum [53] and the virus can be isolated from the brain tissues of infected animals [54], [55]. To date, little has been done to elucidate the molecular mechanism related to the nervous system lesions caused by PRRSV. These results implied that NSP2 might be involved in the process of the nervous system lesions during PRRSV infection. Therefore, a more profound endeavor will be needed to focus on exploring the mechanism of how NSP2 involved in the influence on nervous system.

### The Functions of the Interaction of NSP2 with Host Cellular Proteins BAG6 and AIF1

It has been confirmed that the virus-driven DMVs, which are originated from ER membranes, can be observed in the arterivirus-infected cells [20]–[22]. The DMVs are thought to be associated with the RTCs, which provide suitable environment for viral RNA synthesis and preventing host anti-virus defense system [43]. The Cryo-electron microscopy (cryo-EM) analysis revealed that NSP2 is localized to DMVs^21^, but how the NSP2 is transported to DMVs is still unknown. Out of the 285 identified cellular proteins, 17 proteins were involved in the pathway of protein processing in endoplasmic reticulum, including one interesting protein-BAG6 which we are interested for further analysis. BAG6 is also called as BAT3 (HLA-B-associated transcript 3) or Scythe, which is located in the major histocompatibility complex class ΙΙΙ (MHC ΙΙΙ) of human chromosome 6 [56]. Recently, a series of studies have described that BAG6 is a master regulator of tail-anchored (TA) protein quality control [57]. The TA proteins possess a hydrophobic transmembrane sequence near the C-terminus and hydrophilic N-termini in the cytoplasm [58]. And the newly synthesized TA proteins that are bound by BAG6 have different fates. Some proteins can be delivered to the ER membrane [59], [60], whereas others can be degraded by BAG6-associated ubiquitin-proteasome proteolytic pathway [61], [62]. NSP2 of PRRSV has the similar structure to the TA proteins, with a C-terminal transmembrane (TM) domain and hydrophilic N-termini [12], [13]. In addition, NSP2 possess the de-conjugating activity and the ability to inhibit the polyubiquitination process [23]–[25]. Based on the evidence mentioned above, we speculate that NSP2 is more likely transported to ER membrane by BAG6 complex, instead of being degraded by BAG6 recruited ubiquitin-proteasome system. The interesting issue how PRRSV exploit BAG6 to sustain viral propagation and survival in host cells will be conducted in our future research.

BAG6 is regarded as a functionally diverse protein. Except for the ones mentioned above, BAG6 is also a regulator of apoptosis through interacting with Reaper [57], [63], [64]. Recent studies have implicated that BAG6 play a dual role in regulating apoptosis: promoting or inhibiting [57]. Also BAG6 has been known to enhance the replication of the African swine fever virus (ASFV) through modulating apoptosis of host cells [65].

Among the identified proteins of the interactome profile of NSP2, another apoptosis pathway-associated protein (AIF1) was recognized as well in our study (Figure 6). The interactions between PRRSV NSP2 and porcine BAG6 or AIF1 were further confirmed by Co-IP (Figure 7). As well known, AIF1 is a mitochondrial flavoprotein contributing to caspase-independent cell death [66], and it also plays a key role in mediating poly (ADP-ribose) polymerase-1 (PARP-1)-dependent cell death [67], [68]. The pivotal process of AIF1-inducing apoptosis is that mitochondrial AIF1 translocates to nucleus and induces chromatin condensation and large-scale DNA fragmentation [69]. More and more experiments have proven that the caspase-dependent apoptotic pathway is activated by PRRSV infection [70]–[72] and NSP2 play a crucial role in inducing apoptosis [73]. Here, based on the interaction between NSP2 and AIF1 we suggest that the caspase-independent apoptosis may be involved in PRRSV-driven apoptosis.

The relationship between BAG6 and AIF1 in apoptosis has been investigated previously. Through the interaction with BAG6, AIF1 promotes the phosphatidylserine exposure on the surface of apoptotic cells and subsequent macrophages clearance of these cells [74]. Another study indicated that BAG6 interact with AIF1 and the BAG6 regulate the stability of AIF1 to increases the AIF1-driven apoptosis [75]. In the present research, our results showed that both BAG6 and AIF1 could interact with NSP2 of PRRSV (Figure 6 and 7). These findings not only generate new view on the complicated molecular mechanisms in PRRSV-driven apoptosis, but also provide new targets for future research.

## Conclusion

In the present study, 285 host cellular proteins with high Confidence Icons were identified to interact with the NSP2 in PRRSV-infected cells, by a 3xMyc tagged-recombinant virus, coupled with immunoprecipitation and LC/MS-MS method. According to the Gene Ontology and enriched KEGG Pathway analysis, the identified proteins were assigned to different subcellular locations and functional classes. The interactome profile of NSP2 with the host cellular proteins was first drawn to gain a functional insight into the host-virus proteins interaction. Moreover, two interested host cellular proteins BAG6 and AIF1, which may involve in the transportation of NSP2 to ER or PRRSV-driven apoptosis, were confirmed to interact with NSP2 by Co-IP and Western blot. In summary, our results not only provide valuable information for understanding the roles of NSP2 in the replication and pathogenesis of PRRSV, but also offer novel host cellular protein targets for elucidating the associated molecular mechanisms of the interaction between host cellular proteins and viral proteins in regulating the viral replication.

## Supporting Information

Table S1 **The list of proteins interacting with Nsp2 during PRRSV infection.**
(XLS)

Table S2 **The annotation of proteins interacting with Nsp2 during PRRSV infection using Gene Ontology.**
(XLS)

Table S3 **The list of the enriched KEGG Pathways of the PRRSV Nsp2 interacting proteins.**
(XLS)
